# The clinical efficacy of ozone combined with steroid in the treatment of discogenic low back pain: a randomized, double-blinded clinical study

**DOI:** 10.3389/fneur.2023.1078111

**Published:** 2023-08-10

**Authors:** Xiao-hui Yang, Xiao-hui Liu, Yun-gai Ma, Jia-xing Fan, Xiao-long Ma, Guan-ying Zhuang, Zhan-min Yang

**Affiliations:** Pain Clinic of Anesthesiology Department, Aerospace Center Hospital, Beijing, China

**Keywords:** per-paravertebral, double-blinded clinical study, pain VAS scores, adverse events, discogenic low back pain

## Abstract

**Objective:**

This randomized double-blinded clinical study is to investigate the clinical efficacy of per-paravertebral disk ozone injection combined with steroids in the treatment of patients with chronic discogenic low back pain (CDLBP).

**Methods:**

Group A (*N* = 60) received a per-paravertebral injection of a steroid mixture of 10 mL with pure oxygen 20 mL, while group B (*N* = 60) received a per-paravertebral injection of a steroid mixture of 10 mL combined with ozone 20 mL (30 μg/mL). Injections were administered once a week for 3 weeks, with a follow-up of 6 months. Clinical outcomes were assessed at week 1, month 3, and month 6 with the help of Visual Analog Scale (VAS) scores and Macnab efficacy evaluation.

**Results:**

The VAS score of both group A (1.65 vs. 6.87, *p* = 0.000) and group B (1.25 vs. 6.85, *p* = 0.000) at week 1 was significantly reduced compared to baseline. The effect was sustained at the 3- and 6-month follow-up periods (*p* < 0.05). Group B had significantly lower VAS scores at month 3 (1.53 vs. 3.82, *p* = 0.000) and month 6 (2.80 vs. 5.05, *p* = 0.000) compared to group A, respectively. Based on Macnab criteria, 95 and 96.7% of patients in groups A and B had good rates “excellent plus good” at week 1, respectively. Good rates were significantly higher in group B at month 3 (91.7 vs. 78.3%, *p* = 0.041) and month 6 (85.0 vs. 68.3%, *p* = 0.031) compared to group A, respectively. No serious adverse events were noted in both groups.

**Conclusion:**

Per-paravertebral injection of steroid and ozone combination resulted in better relief of CDLBP compared to pure oxygen plus steroid.

**Clinical Trial Registration:**

ChiCTR2100044434 https://www.chictr.org.cn/showproj.html?proj=121571.

## Introduction

Discogenic pain secondary to intervertebral disk degeneration is recognized as one of the leading causes of chronic discogenic low back pain (CDLBP). It is a prevalent disorder with an occurrence rate of 26–39% among patients with CDLBP (1, 2). Although most people recover without treatment, 37–54% may still have pain a year later (3). In 5–15% of patients, disk degeneration causes discomfort, while 60–80% have no known cause (4). Such degenerative changes in the disk wall, with the subsequent herniation of the disk core content, eventually result in pressure effects on adjacent neural structures. LBP was found to be the biggest cause of worldwide productivity loss and years lived with disability in 126 countries in a recent assessment of 354 disorders (5). LBP costs the United Kingdom £2.8 billion and the United States $100 billion annually (6, 7).

This leads to the presentation of back pain, which sometimes extends to reach the lower limbs, resulting in a disability of a deficit. Medical therapy [i.e., nonsteroidal anti-inflammatory drugs (NSAIDs) and neuromuscular blockers], physical therapy, and rehabilitation are the main management approaches. Meanwhile, open surgery is commonly approached in such cases with the resection of the material within the herniated disk (8, 9).

As alternatives for open surgery, various minimally-invasive measures have been proposed for CDLBP. These measures include the intradiscal injection of steroids, intradiscal injection of methylene blue, intradiscal electrothermal therapy, disk biacuplasty, intradiscal radiofrequency nucleus ablation, DiscTrode, ramus communicans thermocoagulation, and intradiscal pulsed radiofrequency (10, 11). These methods have shown promising effects; however, their efficacy is not yet confirmed (8, 9, 12, 13).

In terms of invasiveness and associated morbidity, minimally invasive approaches are much preferred over the surgical management of CDLBP. Ozone chemonucleolysis, despite some controversies, several papers (14–16) have proposed its relevant medical functionalities, with significant applications in chronic inflammatory conditions, ischemic disorders, infections, and wound healing. Furthermore, it also has shown promising results for the treatment of discogenic pain in recent years (4, 11, 17, 18). *In vivo*, local injection of medical ozone increased TNF-α, IL1β, and IFN-around the disk, suggesting that medical ozone affects the extracellular matrix, shrinking and decompressing the surrounding neurons. Low back pain and sciatica may diminish along with lactic acid and inflammatory cytokines (19).

Ozone chemonucleolysis involves the injection of ozone gas into the intervertebral disk under either fluoroscopic or computed tomography (CT) guidance. Ozone reduces the volume of the disk content by oxidizing the core proteins of the nucleus pulposus (20, 21). Several studies have investigated the efficacy of ozone treatment (either alone or in combination with other drugs) in reducing the degree of low back pain (22–25). Compared to other therapeutic approaches (steroid alone, sham procedure, steroid plus anesthetic, and global postural re-education), medical ozone resulted in a significant reduction in pain at 3 weeks, 1 month (26), and 6 months of treatment (27–30). In this context, we conducted this clinical trial to determine the 6-month clinical efficacy of per-paravertebral injection of medical ozone combined with steroids in the treatment of CDLBP.

## Materials and methods

This randomized, double-blinded clinical study was approved by the Institutional Review Board (IRB)-Ethics Committee of Human Research at the Central Hospital of China Aerospace Corporation, Beijing, China with the registry number 2015QN01 (Registration Date: January 15^th^, 2015) and by the Clinical Trial Center with the registry number ChiCTR2100044434 (Registration Date: March 18, 2021). All eligible individuals were asked to provide written informed consent prior to participation in our study. This study was conducted in line with the guidelines declared by the Helsinki Declaration.

All patients (age ≥ 18 years) were admitted to Aerospace Center Hospital from January 2016 to March 2019. The patients, who had following all inclusion criteria, would be enrolled in this clinical trial: 18–80 years of age; low back pain durated or failed medical therapy, rest, and physiotherapy more than 3 months; sitting and/or lumbar flexion aggravated low back pain; magnetic resonance imaging (MRI) indicated lumbar intervertebral disk pathological changes (such as annular fissures, extrusion, bulging or protrusion, etc.), pathological lesion was found in singal level of intervertebral disc. Excluded patients included those who had a spinal fracture, inflammatory disease, malignancy, facet joint syndrome, previous spinal surgeries, radicular pain, neurological disorders, severe concurrent systemic disease, mental illnesses, coagulation disorders, current anticoagulant therapy, or pregnancy and breastfeeding. CT and Magnetic Resonance Imaging (MRI) were performed before treatment to eliminate the possibility of a space-occupying lesion.

Recruited patients were then randomly assigned to two groups: the control arm (group A) and the treatment arm (group B). Patients in group A received steroid combined with pure oxygen, while patients in B received medical ozone (O2-O3) combined with steroid.

### Sample size calculation

According to our small sample-sized trial, the effective rate of the experimental group is 90%, and the effective rate of the control group is 70%. Assuming that the type I error is 0.05 and the degree of power is 0.8, the estimated sample size in each group (with 1: 1 randomization) was 60 cases. Sample size calculation was estimated according to the following formula, noting that p2 is the effective rate of the test group, p1 is the effective rate of the control group, Z0.05 = 1.96, and Z0.2 = 0.84:

$$n=\frac{{\left(Z_{1-\alpha }+Z_{1-\beta }\right)}^{2}\times [p_{1}\times \left(1-p_{1}\right)+p_{2}\times (1-p_{2})]}{{\left(p_{2}-p_{1}\right)}^{2}}.$$

### Blinding and randomization

Both the investigator, who carried out all operations and assessed pain outcomes, and recruited participants were blinded to the original treatment for the entire period of the study. Recruited subjects were randomized 1:1 to the combined steroid and ozone therapy arm or the control (steroid with pure oxygen) arm for the 3 weeks of treatment using a validated verified randomization program.

### Treatments and procedures

All procedures were carried out in the operation room by the same operation under moderate sedation with local anesthesia of 0.5% lidocaine. Patients in both groups were in the lateral position; the upper spine of the responsible intervertebral disk was selected at a distance of 2–2.5 cm to the puncture point. The area was then prepared with antiseptic lotions. A 21G lumbar puncture needle was then inserted vertically into pinpoint hit ipsilateral vertebral plate lateral lamina. The needle was moved 1–1.5 cm deep into the skin, and the angle of the puncture was adjusted. When the needle was inserted into the disk, a certain resistance was felt. Prior to injection, it was confirmed that the needle tip was situated in the nucleopulposus under the anteroposterior (AP) and lateral C-arm views to overcome injection into the outer annulus. When the resistance to gas injection disappeared, indicating that the intervertebral gap was reached, the assigned treatment was injected. The same method was approached in both groups. After the procedure was done, patients were observed for 15 min to ensure that there were no adverse reactions to the injected drugs. The frequency of injection of the assigned treatment was once a week, with a total of three injections being allowed (safe therapeutic limits).

Patients in group A received a mixture of steroid therapy (40 mg triamcinolone acetonide 1 mL, 0.5% lidocaine 2 mL, and 0.9% physiological saline 7 mL) combined with pure oxygen 20 mL. On the other hand, patients in group B were injected with the same mixture of steroid therapy and medical ozone (O_2_-O_3_) at a volume of 20 mL with a concentration of 30 μg/mL. Ozone injection was administered with the help of an ozone generator (Ozomed Basic; Kastner-Praxisbedarf GmbH, Rastatt, Germany).

### Follow-up and assessments

During the postoperative period, all patients were followed-up regularly either through the phone or as an outpatient follow-up visit. The efficacy of administered interventions was assessed using the pain visual analog scale (VAS) score and the Macnab grading method of the curative effect of each therapy at 1 week (short-term), 3 months (middle-term), and 6 months (long-term). We then used the term “good rate” to indicate the efficacy of both interventions according to the Macnab criteria. The good rate combined the rates of both “excellent to good” responses. Patients were given thorough training on how to record the pain score, functional disability, and any potential complications or limitations during the follow-up period. All scores were compared to the baseline score prior to performing the study procedures. The safety of administered interventions was evaluated by recording the incidence of adverse events.

### Statistical analysis

The intention-to-treat principal was applied for data analyses. The Statistical Package for Social Sciences (SPSS-Version 19.0) software was used for running the statistical analyses. Descriptive analysis was performed for patients’ demographic and baseline clinical data. Repeated measures ANOVA (a parametric test) was used to compare the pain VAS scores between the pre-treatment and the post-treatment time-points. Multifactor repeated measurement variance analysis was used to estimate the differences between the group comparisons. The Pearson chi-square test was used to evaluate the extent of significance in pain relief and the functional status of patients after treatment and during follow-up. A value of *p* < 0.05 was considered to be statistically significant in all analyses.

## Results

### Baseline characteristics

**Figure 1:**
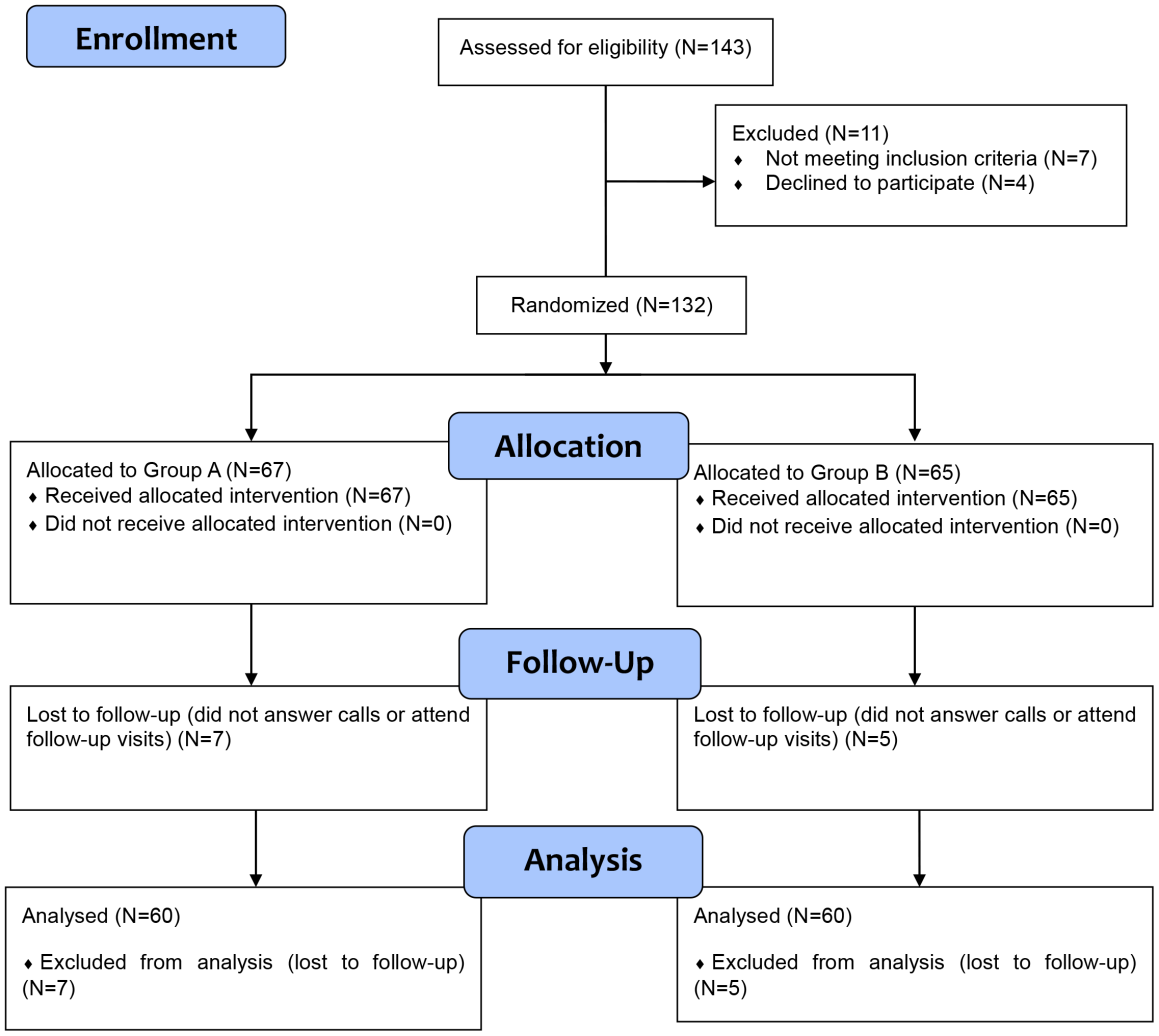
The COSORT flow diagram of this clinical study.

Among the 132 who were included in the study, 67 were allocated to group A, and 65 were allocated to group B. All patients received the allocated interventions; however, seven and five patients in group A and group B were lost to follow-up, respectively. These cases were excluded from the analysis due to unavailable data. The CONSORT flow diagram of our trial is presented in Figure 1. A total of 120 patients were included in the final analysis step. No significant differences were noted between both groups as regards baseline demographic and clinical characteristics (Table 1).

**Table 1:** Baseline demographic characteristics of included participants.

Variable	Sub-group	Group A (67 patients)	Group B (65 patients)	X^2^/*F* value	*p* value
Age (years): Mean ± SD					
		62.9 ± 11.7	66.6 ± 11.2	1.847	0.067
Gender: *N* (%)					
	Male	23(34.33%)	20 (30.77%)	0.190	0.663
	Female	44 (65.67%)	45 (69.23%)		
Duration of symptoms (months): Mean ± SD					
		11.5 ± 3.6	11.2 ± 4.1	0.094	0.76
Lesion Section: *N* (%)					
	L1–L2	2 (2.99%)	1 (1.54%)	2.215	0.696
	L2–L3	1(01.49%)	3 (4.62%)		
	L3–L4	4 (5.97%)	6 (9.23%)		
	L4–L5	42 (62.69%)	39(60.00%)		
	L5–S1	18 (26.877%)	16(24.62%)		

SD, standard deviation; N, number; X2, Chi-Square test; L, lumbar; S, sacral; Group A, patients who received steroid and pure oxygen; and Group B, patients who received steroid combined with ozone therapy.

### Efficacy of administered interventions

In terms of intra-group comparisons, both group A (1.63 ± 1.31 vs. 6.19 ± 1.13, *p* < 0.001) and group B (1.24 ± 0.99 vs. 6.57 ± 1.61, *p* < 0.001) revealed a significant reduction in mean pain VAS scores compared to baseline (before treatment) at week 1, respectively. This mean VAS score remained significantly lower than baseline, in both groups, at months 3 and 6 of assessment (*p* < 0.001), respectively (Table 2). Meanwhile, 95 and 96.7% of patients in group A and group B had “good rates” according to Macnab criteria at week 1, respectively (Table 3). However, the percentage of “good rate” was noted to decrease in group A (78.3 vs. 68.3%) and group B (91.7 vs. 85.0%) at 3 and 6 months, respectively.

**Table 2:** VAS scores before and after treatment in studied groups ( $\bar{x}$ ± s).

Group	Number	Before treatment	After treatment			*F* value	*p* value
			1 week	3 months	6 months		
Group A	67	6.19 ± 1.13	1.63 ± 1.31^a^	3.81 ± 1.29^a^	5.04 ± 1.26^a^	168.832	0.000^*^
Group B	65	6.57 ± 1.61	1.24 ± 0.99^a^	1.52 ± 1.15^ab^	2.82 ± 1.20^ab^	276.898	0.000^*^
*F* value		1.553	3.619	104.911	97.23		
*p* value		0.123	0.058	0.000^*^	0.000^*^		

* Highly significant.

^a^Compared with before treatment (*p* < 0.05); ^b^compared with group A (*p* < 0.05). Group A, patients who received steroid plus pure oxygen; Group B, patients who received steroid plus ozone.

**Table 3:** Comparison of Macnab efficacy evaluation after treatment in two groups.

Groups		Number	Excellent	Good	Fair	Poor	Good rate^*^	χ^2^	*p*
Group A									
	1 week	60	51	6	3	0	57 (95.0%)	NA	NA
	3 months	60	36	11	8	5	47 (78.3%)	NA	NA
	6 months	60	33	8	11	8	41 (68.3%)	NA	NA
Group B									
	1 week	60	52	6	2	0	58 (96.7%)	0.209	0.648
	3 months	60	47	8	4	1	55 (91.7%)^a^	4.183	0.041
	6 months	60	45	6	5	4	51 (85.0%)^a^	4.658	0.031

χ^2^, Chi-square test; *p*, *p* value; ^a^comparison with group A (*p* < 0.05). Group A, patients who received steroid plus pure oxygen; Group B, patients who received steroid plus ozone. ^*^Good rate is the rate of cases who had excellent and good MacNab scores. Data are presented as numbers and percentages.

As regard between-group comparisons, the reduction in mean VAS scores in group B was significantly more pronounced than that of group A at month 3 (1.52 ± 1.15 vs. 3.81 ± 1.29, *p* < 0.001), respectively. Similarly, at 6 months of assessment, group B had significantly lower mean VAS scores compared to group A (more pronounced effect), with values of (2.82 ± 1.20 vs. 5.04 ± 1.26, *p* < 0.001), respectively (Table 2). As regard the Macnab criteria, we noted that patients in group B (combined steroid and ozone) had significantly higher “good rates” at 3 months (91.7 vs. 78.3%, *p* = 0.041) and 6 months (85.0 vs. 68.3%, *p* = 0.031) of assessment compared to group A, respectively (Table 3). This indicates that the efficacy of medical ozone and steroid therapy was more pronounced in group B than group A at 3 and 6 months of assessment.

### Safety profile of administered interventions

There were no serious adverse events recorded in both groups. Two patients in this study experienced numbness in the lower limbs, which disappeared within 2 h. On the other hand, three patients in group B had mild distended pain when they were injected with ozone.

## Discussion

This clinical study assessed the efficacy and safety profile of medical ozone treatment (O^2^-O^3^) for the treatment of low back pain of discogenic origin. A total of 120 were included in this trial, where 60 patients received 20 mL pure oxygen combined with steroid therapy (control group), and 60 patients received 20 mL ozone (30 μg/mL) combined with steroid (intervention group) for the treatment of CDLBP. The mean age of our population is 69.2 years, and L4-L5 was the most commonly affected spinal segment in 62.5%. All patients were followed up at 1 week, 3 months, and 6 months after treatment. We used the pain VAS score to determine the difference in pain at different follow-up time-points compared to baseline. In this study, both interventions resulted in a significant reduction in VAS scores at week 1, month 3, and month 6 compared to baseline. However, the mean pain VAS score was significantly lower (more efficacious) in the medical ozone group compared to the pure oxygen group at month 3 (1.52 vs. 3.81, *p* < 0.001) and month 6 (2.82 vs. 5.04, *p* < 0.001), respectively.

Many research studies have been conducted to estimate the efficacy of medical ozone treatment in relieving low back pain (LBP). In 2015, the randomized double-blinded controlled trial of Perri et al. (27) was published. This trial investigated the efficacy of intradiscal and intraforaminal injection of 10 mg ozone (28 μg/mL) combined with steroid and anesthetic therapy compared to the combined steroid and anesthetic therapy alone among 154 patients (77 cases in each group). Inconsistent with our findings, the authors found that both groups had a similar reduction in pain VAS score; however, patients who received the ozone intervention had significantly lower pain scores at 6 months compared to the control group. This conflicting observation could be related to the difference in the protocol used, the variation in ozone concentration and dose, and the difference in the route of application. In 2018, Rahimzadeh et al. (31) conducted a 12-month trial to compare the efficacy of percutaneous intradiscal injection of 6 mL ozone (30 μg/mL) to laser disk decompression (control) among 40 patients (20 in each group) with low back pain due to intervertebral disk herniation. No significant differences regarding mean VAS scores were noted between both groups at 1, 3, 6, and 12 months. Meanwhile, the authors noted that ozone therapy resulted in better efficacy in relieving pain (significantly lower mean Oswestry Disability Index) compared to laser therapy at 3, 6, and 12 months (*p* < 0.05). On the other hand, Paoloni et al. (28) recruited 60 patients to determine the efficacy of 20 mL intramuscular per-paravertebral ozone (20 mg/mL) compared to the Sham procedure (control) for the treatment of LBP. It was noted that both groups had nearly similar VAS scores at 2, 4, and 6 weeks. However, ozone showed superiority over the sham procedure in relieving pain at 3 and 6 months, which goes in line with our findings.

In our study, the majority of patients in the ozone group (86.7%) and the pure oxygen group (85%) had excellent outcomes, based on the Macnab criteria, in the first week of assessment. For the purposes of determining the most efficacious intervention, we combined the “excellent” cases and the “good” cases, which were then divided by the total sample size to get the “good rate,” which would be indicative of the efficacy of either intervention in relieving pain. Both the ozone and the pure oxygen groups had similar good rates at week one (96.7 vs. 95%, *p* > 0.05), respectively. However, we noted that the “good rate” was significantly higher in the ozone group at month 3 (91.2 vs. 78.3%, *p* = 0.041) and month 6 (85 vs. 68.3%, *p* = 0.031) compared to the control arm, respectively. This indicates that while both groups witnessed improvement in pain outcomes compared to baseline, the per-paravertebral injection of ozone therapy (combined with steroid) had superior efficacy in the long-term (3 and 6 months) compared to pure oxygen and steroid therapy. In the same context, Zambello et al. conducted a trial to investigate the efficacy of 5 mL per-paravertebral ozone injection (10–20 μg/mL) compared to the epidural injection of 80 mg triamcinolone (control) among 351 patients. The authors noted that the percentage of patients who had good rates of “excellent plus good outcomes” based on Macnab criteria was significantly higher in the ozone group at 3 weeks (88.2 vs. 73%, *p* < 0.05) and 6 months (77.7 vs. 55%, *p* < 0.0%) of treatment, respectively. This goes in line with our findings.

Medical ozone treatment is commonly recognized as a procedure with a low-risk of complications (32), and thus, ozone injections are approached in patients with contraindications for surgery or as an exploratory pain relief therapy prior to surgical interventions (28, 33). Even though ozone is perceived as a potentially-toxic agent, very few research studies have actually reported on the complications associated with this therapy. These complications are majorly generic adverse effects, including insomnia, itching, papules are the infiltration point, gastritis, an increase in heart rate, hot flushed, and tachycardia (3, 26). In our study, no serious adverse events were noted in both groups. Three patients in the ozone treatment arm reported mild distended pain upon injection of ozone, and two patients reported numbness of the lower limb. Noteworthy, a limited number of studies explicitly reported on the adverse effects of ozone therapy in treating low back pain (26, 28, 29). In two of the aforementioned trial, no complications were demonstrated in either group (ozone vs. control), while in one trial, a low incidence of complications was noted with no significant difference between ozone treatment and the non-ozone control group. However, neurosurgeons should give significant attention to ozone therapy as some serious infectious events, related to ozone infiltration, have been demonstrated in several studies (34, 35).

Our clinical study gives a helpful insight into the treatment of patients with CDLBP through medical ozone. Both ozone and pure oxygen (combined with steroid) resulted in significant improvement in pain compared to baseline; however, the effect was significantly more pronounced in the ozone group, with minimal and non-serious complications in the short- and long-term. Even though multiple studies have been conducted to determine the clinical efficacy of medical ozone therapy in relieving pain among patients with low back pain of multiple origins, the majority of studies did not report on the number of losses to follow-up and their management and the blinding and randomization methods. Therefore, more robust randomized placebo-controlled trials are still warranted to confirm these findings.

Our study has some strength points including focusing on all three periods of follow-up (3 weeks, 1 month, and 6 months), which was significantly lacking in this area. Furthermore, this study is a special addition to improving the quality of life of patients. However, a number of limitations need to be stated. The control group were not recruited and compared with CDLBP group. Affacts and safety of ozone combined with steroid treatment for healthy people could not be evaluated. Besides, this clinical trial was practiced in a single hospital which could induce statistical bias and increased response variability.

## Conclusion

The per-paravertebral injection of medical ozone, combined with steroids, provides excellent pain relief in patients with CDLBP. It is considered an effective and safe treatment option for such patients. Even though pure oxygen and ozone provide improvement in pain compared to baseline, pain relief is more pronounced in ozone treatment with minimal, non-serious side-effects.

## Data availability statement

The original contributions presented in the study are included in the article/supplementary material, further inquiries can be directed to the corresponding author.

## Ethics statement

The studies involving human participants were reviewed and approved by the Institutional Review Board (IRB)-Ethics Committee of Human Research at the Central Hospital of China Aerospace Corporation (2015QN01). The patients/participants provided their written informed consent to participate in this study.

## Author contributions

X-hY wrote the manuscript. X-hL, Y-gM, and J-xF collected the data. X-lM, G-yZ, and Z-mY analyzed the data. All authors contributed to the article and approved the submitted version.

## Conflict of interest

The authors declare that the research was conducted in the absence of any commercial or financial relationships that could be construed as a potential conflict of interest.

## Publisher’s note

All claims expressed in this article are solely those of the authors and do not necessarily represent those of their affiliated organizations, or those of the publisher, the editors and the reviewers. Any product that may be evaluated in this article, or claim that may be made by its manufacturer, is not guaranteed or endorsed by the publisher.
